# Genome subtraction for the identification of potential antimicrobial targets in *Xanthomonas oryzae* pv. *oryzae* PXO99A pathogenic to rice

**DOI:** 10.1007/s13205-013-0131-7

**Published:** 2013-04-04

**Authors:** V. Keshri, Dhananjaya P. Singh, R. Prabha, A. Rai, A. K. Sharma

**Affiliations:** 1 National Bureau of Agriculturally Important Microorganisms, Indian Council of Agricultural Research, Kushmaur, Maunath Bhanjan, 275101 UP India; 2 Indian Agricultural Statistical Research Institute, Indian Council of Agricultural Research, Library Avenue, Pusa, New Delhi, 110012 India

**Keywords:** *Xanthomonas oryzae*, Rice, Pathogen, Essential genes, Subtractive genomics

## Abstract

In pathogenic bacteria, identification of essential proteins which are non-homologous to the host plants represents potential antimicrobial targets. We applied subtractive genomics approach for the identification of novel antimicrobial targets in *Xanthomonas oryzae* pv. *oryzae* PXO99A, the causative agent of bacterial blight in rice. Comparative analysis was performed through BLAST available with the NCBI. The analysis revealed that 27 essential protein sequences out of 4,988 sequences of *X. oryzae* pv. *oryzae* PXO99A are non-homologous to *Oryza sativa*. Subsequent analysis of 27 essential proteins revealed their involvement in different metabolic activities such as transport activity, DNA binding, structural constituent of ribosome, cell division, translation, and plasma membrane. These 27 proteins were analyzed for virulence and novelty and out of 27, three essential non-homologous proteins were found to be the novel antimicrobial targets.

## Introduction

Generation of vast genomic data from prokaryotic whole genome projects in the recent years has opened new avenues for finding out novel drug targets in microbes (Buysse 2001). Genome sequences of pathogenic microbes provided tremendous information which is now facilitating in silico identification and characterization of potential therapeutic targets and virulence factors of pathogens (Amineni et al. 2010; Dutta et al. 2006; Miesel et al. 2003). Potential therapeutic targets should be an essential component of a particular metabolic pathway in a pathogen, should be adequately selective to yield a drug that is specific against the pathogen and should possess no homolog within the host system so that the designed lead molecule can act against the functionality of the pathogen only but not against the host. Subtractive genomics approach entwined with bioinformatics can find out optimal targets related to previously unknown cellular functions in microbes based on the understanding of relatively similar biological processes in pathogens and hosts (Vetrivel et al. 2011; Koteswara et al. 2010; Sakharkar et al. 2004). Using this approach, a number of potential drug targets have been identified for bacterial pathogens of humans (Barh et al. 2011).

Search for antibacterial targets in bacteria pathogenic to plants has remained an untouched area of in silico research, although there exists tremendous scope for likely work with the appearance of a large volume of data sets coming out from the whole genome sequencing projects of the phytopathogenic bacteria. *Xanthomonas oryzae* pv. *oryzae*, a gamma-proteobacteria is an important pathogen of rice (Swings et al. 1990) causing bacterial leaf blight (Niño-Liu et al. 2006) or bacterial blight (Salzberg et al. 2008). High-yielding rice cultivars are more susceptible to the disease that leads to wilting of seedlings, yellowing and drying of leaves and yield loss. Besides physical disease management practices including sanitation, seed treatment with bleaching powder (10 μg/ml) and zinc sulfate (2 %) are reported to reduce the disease but chemical control using antibiotics has only limited success (Rice Knowledge Bank 2009). Continuously increasing resistance among the pathogens towards antibiotics has generated the need for searching novel antimicrobial targets in pathogenic bacteria that may lead to the development of novel antimicrobial agents.

*Xanthomonas oryzae* pv. *oryzae* strain PXO99A is virulent towards many rice varieties representing diverse genetic sources for resistance and needs novel antimicrobials for reducing leaf blight resistance and increasing rice yield. Complete genome sequences of different strains of *X. oryzae* pv. *oryzae* like PXO99A (Salzberg et al. 2008), KACC10331, and MAFF311018 (Triplett et al. 2011) facilitated in-depth comparative genomic analyses. Genome comparison indicated that strain PXO99A contains various virulence-associated transcription activator-like effector genes and possesses a minimum of 10 major chromosomal rearrangements in comparison to the other strains KACC10331 and MAFF311018 (Salzberg et al. 2008). Looking into the practical implications of such work, we applied subtractive genomics approach to identify novel protein targets that encode pathogenicity in *X. oryzae* pv. *oryzae* PXO99A and help in finding out novel antimicrobial targets to develop potential antimicrobial agents against this important disease of rice.

## Results and discussion

Computational approaches have been applied to identify essential genes in prokaryotes. We reported the identification of essential genes as the potential antibacterial targets in plant pathogenic bacteria *X. oryzae* pv. *oryzae*. The approach was based on sequence alignment of proteins downloaded from the NCBI (Table 1) and database of essential genes (DEG). *X. oryzae* pv. *oryzae* PXO99A, *O. sativa* and database of essential genes (DEG) of prokaryotes contain 4,988, 21,342 and 7,643 protein sequences, respectively. Our results revealed that out of 4,988 proteins in *X.oryzae* pv. *oryzae* PXO99A, 406 unique sequences did not resulted in any hits (no hits found) and did not align with any sequence of *O. sativa*. The result is in agreement with the earlier reports (Jacobs et al. 2003, Sakharkar et al. 2004) who reported classified 300–400 essential genes in another bacteria *P. aeruginosa.* When non-homologous 406 sequences were aligned (two-way BLAST) against prokaryotic essential protein sequences of DEG with an e-value cutoff of 10^−10^ for determination of their essentiality, 27 sequences were found essential for the pathogen. Further functional categorization based on the respective gene description or name of these proteins revealed that in the pathogen, these proteins might be considered as unique and linked with the essential metabolic pathway. All these 27 protein sequences were related to different functional cellular properties such as transport activity, DNA binding, structural constituent of ribosome, cell division, translation, plasma membrane and membrane protein (Table 2).

**Table 1:** Genomic data (NCBI 2012) of *Xanthomonas oryzae* pv. *oryzae* PXO99A

Organism	*Xanthomonas oryzae* pv. *oryzae* PXO99A
Taxonomy ID	360094
Chromosome	Single circular
Protein	4,988
Protein coding genes	5,083
Protein coding genes not found in other strains (KACC10331 or MAFF311018)	87
Genome size	5,240,075 bp
GC %	63.6

**Table 2:** Most probable antibacterial targets in *Xanthomonas oryzae* pv. *Oryzae*

GI/accession number	Description
gi|188574599|ref|YP_001911528.1|	TetR family transcriptional regulator
gi|188574639|ref|YP_001911568.1|	Twin arginine-targeting protein translocase TatC
gi|188574650|ref|YP_001911579.1|	TonB-dependent receptor
gi|188574873|ref|YP_001911802.1|	Isocitrate dehydrogenase
gi|188575043|ref|YP_001911972.1|	Monovalent cation/H+ antiporter subunit A
gi|188575296|ref|YP_001912225.1|	Cell division protein FtsW
gi|188575690|ref|YP_001912619.1|	Signal protein with PAS(PAC), GGDEF and EAL domains
gi|188575924|ref|YP_001912853.1|	Carbon storage regulator
gi|188576015|ref|YP_001912944.1|	Chemotaxis signal transduction protein
gi|188576081|ref|YP_001913010.1|	AcrB protein
gi|188576225|ref|YP_001913154.1|	Argininosuccinate lyase
gi|188576521|ref|YP_001913450.1|	Transposase
gi|188577176|ref|YP_001914105.1|	Lipid-A-disaccharide synthase
gi|188577531|ref|YP_001914460.1|	TonB-dependent outer membrane receptor
gi|188577685|ref|YP_001914614.1|	NADH dehydrogenase subunit L
gi|188577690|ref|YP_001914619.1|	NADH dehydrogenase subunit G
gi|188577693|ref|YP_001914622.1|	NADH dehydrogenase subunit D
gi|188577770|ref|YP_001914699.1|	Protein TldD
gi|188578034|ref|YP_001914963.1|	TonB-dependent outer membrane Receptor
gi|188578050|ref|YP_001914979.1|	cob(I)alamin adenosyltransferase
gi|188578410|ref|YP_001915339.1|	Sulfate ABC transporter permease
gi|188578579|ref|YP_001915508.1|	TonB-dependent outer membrane receptor
gi|188578592|ref|YP_001915521.1|	General secretion pathway protein F
gi|188578888|ref|YP_001915817.1|	Chromosome partitioning protein
gi|188579158|ref|YP_001916087.1|	Xylosidase
gi|188579258|ref|YP_001916187.1|	50S ribosomal protein L34
gi|229358035|ref|YP_001915183.2|	50S ribosomal protein L31

The KEGG GENES database which is a resource for cross-species annotation of all available genomes by KEGG orthology (KO) system, classified all 27 essential genes of *X. oryzae* pv. *oryzae* into different categories according to their involvement in different metabolic pathways (Table 3). Metabolic pathway analysis of essential proteins revealed that majorly three genes are involved in oxidative phosphorylation, three in nitrogen metabolism, two in bacterial secretion system, one in glutathione metabolism, one in arginine and proline metabolism and one each in bacterial chemotaxis and protein export besides several others that are involved in different essential pathways (Table 4). Some of these proteins directly contribute to the basic primary metabolic mechanisms like carbon fixation, phosphorylation, amino acid biosynthesis, citric acid cycle, nitrogen metabolism, etc. However, certain essential proteins like those encoding ABC transporters (1), bacterial chemotaxis (1), protein export (1) and secretion systems (2) found in the pathogen are, in one or the other way, linked to the pathogenicity, virulence factor, nutrient mobilization and uptake and motility of the organisms (Maranhão et al. 2009; Rodriguez and Smith 2006; Stergiopoulos et al. 2003). Virulent/non-virulent properties predicted through support vector machine (SVM) approach revealed three virulent proteins (accession number: YP_001911579.1, YP_001913450.1, YP_001914963.1) leading to the assumption that these three essential proteins could have important role in the normal functioning of the pathogen within the host. Thus, these proteins can be viewed as novel targets because of their non-significant similarity with DrugBank targets and can be used for the development of antimicrobials. These three proteins [YP_001911579.1 (TonB-dependent receptor), YP_001913450.1 (Transposase) and YP_001914963.1 (TonB-dependent outer membrane receptor)] are very critical due to their presence in the outer membrane of the pathogens.

**Table 3:** KEGG orthology of all essential potential antimicrobial targets

GI/accession number	KO	KO description
gi|188574599|ref|YP_001911528.1|	–	–
gi|188574639|ref|YP_001911568.1|	K03118	Sec-independent protein translocase protein TatC
gi|188574650|ref|YP_001911579.1|	–	–
gi|188574873|ref|YP_001911802.1|	K00031	Isocitrate dehydrogenase
gi|188575043|ref|YP_001911972.1|	K05559	Multicomponent K+:H+ antiporter subunit A
gi|188575296|ref|YP_001912225.1|	K03588	Cell division protein FtsW
gi|188575690|ref|YP_001912619.1|	–	–
gi|188575924|ref|YP_001912853.1|	K03563	Carbon storage regulator
gi|188576015|ref|YP_001912944.1|	K03408	Purine-binding chemotaxis protein CheW
gi|188576081|ref|YP_001913010.1|	K03296	Hydrophobic/amphiphilic exporter-1 (mainly G-bacteria), HAE1 family
gi|188576225|ref|YP_001913154.1|	K01755	Argininosuccinate lyase
gi|188576521|ref|YP_001913450.1|	–	–
gi|188577176|ref|YP_001914105.1|	K00748	Lipid-A-disaccharide synthase
gi|188577531|ref|YP_001914460.1|	–	–
gi|188577685|ref|YP_001914614.1|	K00341	NADH-quinone oxidoreductase subunit L
gi|188577690|ref|YP_001914619.1|	K00336	NADH-quinone oxidoreductase subunit G
gi|188577693|ref|YP_001914622.1|	K00333	NADH-quinone oxidoreductase subunit D
gi|188577770|ref|YP_001914699.1|	K03568	TldD protein
gi|188578034|ref|YP_001914963.1|	K02014	Iron complex outermembrane recepter protein
gi|188578050|ref|YP_001914979.1|	K00798	Cob(I)alamin adenosyltransferase
gi|188578410|ref|YP_001915339.1|	K02047	Sulfate transport system permease protein
gi|188578579|ref|YP_001915508.1|	K02014	Iron complex outermembrane recepter protein
gi|188578592|ref|YP_001915521.1|	K02455	General secretion pathway protein F
gi|188578888|ref|YP_001915817.1|	K03497	Chromosome partitioning protein, ParB family
gi|188579158|ref|YP_001916087.1|	K01198	Xylan 1,4-beta-xylosidase
gi|188579258|ref|YP_001916187.1|	–	–
gi|229358035|ref|YP_001915183.2|	K02909	Large subunit ribosomal protein L31

**Table 4:** Pathway mapping of all essential potential antimicrobial targets

Pathways	No. of protein
Citrate cycle (TCA cycle)	1
Oxidative phosphorylation	3
Alanine, aspartate and glutamate metabolism	1
Arginine and proline metabolism	1
Glutathione metabolism	1
Starch and sucrose metabolism	1
Amino sugar and nucleotide sugar metabolism	1
Lipopolysaccharide biosynthesis	1
Carbon fixation pathways in prokaryotes	1
Porphyrin and chlorophyll metabolism	1
Nitrogen metabolism	3
ABC transporters	1
Two-component system	2
Bacterial chemotaxis	1
Ribosome	1
Protein export	1
Bacterial secretion system	2
Cell cycle–Caulobacter	1
Peroxisome	1

## Experimental procedure

### Data retrieval

Complete protein sequences of *X. oryzae* pv. *oryzae* PXO99A and *Oryza sativa* were downloaded from NCBI (ftp://ftp.ncbi.nih.gov/genomes/). Essential genes of prokaryotes were downloaded from the DEG (http://tubic.tju.edu.cn/deg/) (Zhang et al. 2004). For comparative analysis, known protein targets existing with the DrugBank were downloaded (Wishart et al. 2008).

### Identification of essential proteins

Complete protein sequence of *X. oryzae* pv. oryzae PXO99A was subjected to BLASTP (Altschul et al. 1990) against *O. sativa* protein sequences to identify gene products of the pathogen. Sequences that did not show any similarity were further subjected to BLASTP with e-value cutoff score of 10^−10^ against all prokaryotic sequences of the DEG (Zhang et al. 2004) to screen out genes that appeared to represent essential genes. Further, biological processes, molecular functions and cellular components have been identified. Complete BLAST alignments were two-way.

### Metabolic pathway analysis

**Fig. 1:**
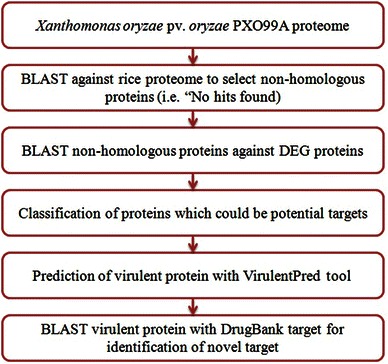
Flow chart of systematic identification of novel targets in *Xanthomonas oryzae* pv. *oryzae* PXO99A

Metabolic pathway analysis was carried out by KAAS (KEGG Automatic Annotation Server) at KEGG for the identification of essential proteins in different pathways. KAAS provides functional annotation of genes by BLAST comparisons against the manually curated KEGG GENES database. The result contains KO assignments and automatically generated KEGG pathways (Moriya et al. 2007). The method for identification of probable antibacterial targets is described in Fig. 1.

### Prediction of virulent proteins

Bacterial virulent protein sequences were predicted through bi-layer cascade support vector machine (SVM) based prediction tool VirulentPred (Garg and Gupta 2008). In the first layer SVM classifiers were trained and optimized with different individual protein sequence features and cascaded to the second layer SVM classifier to train and generate the final classifier. The selected prediction approach for the query were amino acid composition, dipeptide composition, similarity searching, higher order dipeptide composition, PSSM and cascased SVM module.

### Prediction of targets as novel

All protein targets of DrugBank (Wishart et al. 2008) were downloaded and aligned with essential proteins for finding out the significant similarity and dissimilarity that represent non-novel and novel targets, respectively. Sequences were subjected to BLASTP with e-value cutoff score of 10^−4^ and similarity >70 against all the experimental drug targets.

## References

[CR1] Altschul SF, Gish W, Miller W, Myers EW, Lipman DJ (1990). Basic local alignment search tool. J Mol Biol.

[CR2] Amineni U, Pradhan D, Marisetty H (2010). In silico identification of common putative drug targets in Leptospira interrogans. J Chem Biol.

[CR3] Barh D, Tiwari S, Jain N, Ali A, Santos AR, Misra AN, Azevedo V, Kumar A (2011). In silico subtractive genomics for target identification in human bacterial pathogens. Drug Dev Res.

[CR4] Buysse JM (2001). The role of genomics in antibacterial target discovery. Curr Med Chem.

[CR5] Dutta A, Singh SK, Ghosh P, Mukherjee R, Mitter S, Bandyopadhyay D (2006). In silico identification of potential therapeutic targets in the human pathogen Helicobacter pylori. In Silico Biol.

[CR6] Garg A, Gupta D (2008). VirulentPred: a SVM based prediction method for virulent proteins in bacterial pathogens. BMC Bioinform.

[CR7] Jacobs MA, Alwood A, Thaipisuttikul I, Spencer D, Haugen E, Ernst S, Will O, Kaul R, Raymond C, Levy R, Chun-Rong L, Guenthner D, Bovee D, Olsen MV, Manoil C (2003). Comprehensive transposon mutant library of *Pseudomonas aeruginosa*. Proc Nat Acad USA.

[CR8] Koteswara RG, Nagamalleswara RK, Phani RK, Aravind S (2010). In Silico identification of potential therapeutic targets in Clostridium botulinum by the approach subtractive genomics. Indian J Bioinform Res.

[CR9] Maranhão FC, Paião FG, Fachin AL, Martinez-Rossi NM (2009). Membrane transporter proteins are involved in Trichophyton rubrum pathogenesis. J Med Microbiol.

[CR10] Miesel L, Greene J, Black TA (2003). Genetic strategies for antibacterial drug discovery. Nat Rev Genet.

[CR11] Moriya Y, Itoh M, Okuda S, Yoshizawa AC, Kanehisa M (2007). KAAS: an automatic genome annotation and pathway reconstruction server. Nucleic Acids Res.

[CR12] Niño-Liu DO, Ronald PC, Bogdanove AJ (2006). *Xanthomonas oryzae* pathovars: model pathogens of a model crop. Mol. Plant Pathol.

[CR13] Rice Knowledge Bank (2009) (http://www.knowledgebank.irri.org/RiceDoctor/information-sheets-mainmenu-2730/diseases-mainmenu-2735/bacterial-leaf-blight-mainmenu-2758.html)

[CR14] Rodriguez GM, Smith I (2006). Identification of an ABC transporter required for iron acquisition and virulence in Mycobacterium tuberculosis. J Bacteriol.

[CR15] Sakharkar KR, Sakharkar MK, Chow VT (2004). A Novel Genomics Approach for the Identification of Drug Targets in Pathogens, with Special Reference to *Pseudomonas aeruginosa*. Silico Biol.

[CR16] Salzberg SL, Sommer DD, Schatz MC, Phillippy AM, Rabinowicz PD, Tsuge S, Furutani A, Ochiai H, Delcher AL, Kelley D, Madupu R, Puiu D, Radune D, Shumway M, Trapnell C, Aparna G, Jha G, Pandey A, Patil PB, Ishihara H, Meyer DF, Szurek B, Verdier V, Koebnik R, Dow JM, Ryan RP, Hirata H, Tsuyumu S, Won Lee S, Seo YS, Sriariyanum M, Ronald PC, Sonti RV, Van Sluys MA, Leach JE, White FF, Bogdanove AJ (2008) Genome sequence and rapid evolution of the rice pathogen *Xanthomonas oryzae* pv. *oryzae* PXO99A. BMC Genomics 9:204 (erratum 9: 534) 10.1186/1471-2164-9-204 PMC2432079 18452608

[CR17] Stergiopoulos I, Zwiers LH, De Waard MA (2003). The ABC transporter MgAtr4 is a virulence factor of *Mycosphaerella graminicola* that affects colonization of substomatal cavities in wheat leaves. Mol Plant Microbe Interact.

[CR18] Swings J, Mooter MVD, Vauterin L, Hoste B, Gills M, Mew TW, Kersters K (1990). Reclassification of the causal agents of bacterial blight (*Xanthomonas camprestris* pv.*oryzae*) and bacterial leaf streak (*Xanthomonas camprestris* pv.*oryzicola*) of rice as pathovars of *Xanthomonas oryzae* (ex Ishiyama) 1922) sp. nov., nom. Rev Int J Syst Bacteriol.

[CR19] Triplett LR, Hamilton JP, Buell CR, Tisserat NA, Verdier V, Zink F, Leach JE (2011). Genomic analysis of *Xanthomonas oryzae* isolates from rice grown in the United States reveals substantial divergence from known *X. oryzae* pathovars. Appl Environ Microbiol.

[CR20] Vetrivel U, Subramanian G, Dorairaj S (2011). A novel in silico approach to identify potential therapeutic targets in human bacterial pathogens. Hugo J.

[CR21] Wishart DS, Knox C, Guo AC, Cheng D, Shrivastava S, Tzur D, Gautam B, Hassanali M (2008). DrugBank: a knowledgebase for drugs, drug actions and drug targets. Nucleic Acids Res.

[CR22] Zhang R, Ou HY, Zhang CT (2004). DEG: a database of essential genes. Nucleic Acids Res.

